# Endometrioma of the Rectus Abdominis Muscle: US Features

**DOI:** 10.5334/jbr-btr.856

**Published:** 2015-09-15

**Authors:** L. Van Camp, K. Mermuys

**Affiliations:** 1 Resident, Department of Radiology, AZ Sint-Jan Bruges, Belgium; 2 Staff Radiologist, Department of Radiology, AZ Sint-Jan Bruges, Belgium

An otherwise healthy 36-year-old woman consulted our department with abdominal wall pain. She had a history of double Cesarean section, and lumbar scoliosis. The pain was described as cramping and soar, intensifying after physical activity. The painful area was located cranially to the right superior pubic ramus, radiating towards the right inguinal region. Abdominal ultrasound revealed a heterogeneous mass located in the rectus abdominis muscle, in proximity of its insertion. Hyperechoic and hypoechoic nodules were present, accompanied by a mild degree of internal vascularization (Fig. A). The mass was well defined, and could be differentiated from the surrounding rectus abdominis muscle. Dimensions were approximately 2.27 × 1.23 × 3.41 centimetres (Figs. B, C). Due to the sonographic features and clinical history, the diagnosis of endometrioma of the rectus abdominis muscle was suggested. Histopathology after surgical excision confirmed this diagnosis later on.

**Figures A–C:**
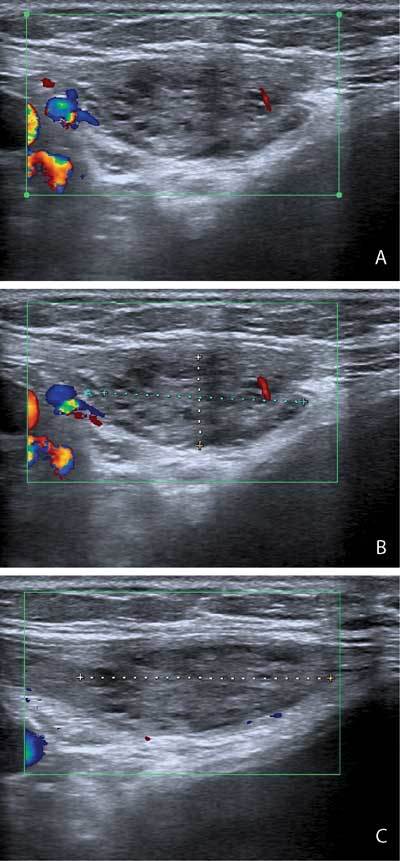


## Comment

Endometrioma in the rectus abdominis muscle is a rare condition. The disease presents in a population of mostly young females of child-bearing age, often with previous history of Cesarean section or gynecological surgery. It is defined as the presence of endometrial tissue outside the uterine cavity. The sonographic appearance is variable. Common sonographic findings are a hypoechoic mass with inhomogeneous echo texture, scattered hyperechoic echoes and irregular margins, as well as solid or cystic lesions. A hyperechoic ring often notes inflammatory alterations. Internal vascularization can be detected in almost every case. Ultrasound is often the first diagnostic tool. Abdominal CT or MR can prove useful in the differential diagnosis. FNAC should be avoided, as spreading of the tissue can occur. It should be noted that neither of the imaging modalities can provide a certain pre-operative diagnosis. The final diagnosis is made by pathological examination after surgical excision of the lesion.

The differential diagnosis can be difficult, and includes ventral hernia, hematoma, lymphadenopathy, lymphoma, lipoma, abscess, subcutaneous or sebaceous cyst, suture granuloma, soft-tissue sarcoma, desmoid tumors, and even metastatic cancer. Surgical excision provides the best therapeutic option. In the absence of a suggesting clinical history, the differential diagnosis can be difficult.

## Competing Interests

The authors declare that they have no competing interests.
